# Pathological impairments induced by interstitial implantation of ^125^I Seeds in spinal canal of banna mini-pigs

**DOI:** 10.1186/1477-7819-10-48

**Published:** 2012-03-05

**Authors:** Zuozhang Yang, Yongqing Xu, Dakuan Yang, Hongpu Sun, Ruilian Zhao, Jin Zhang, Xiaoxue Wang, Hua Jiang, Lie Xu, Jinlei Zhang

**Affiliations:** 1 Department of Orthopaedics, Kunming General Hospital of Chengdu Military Command, Kunming, Yunnan 650118, P. R. China; 2 Department of Orthopedics, Tumor Hospital of Yunnan Province, The Third Affiliated Hospital of Kunming Medical University, Kunming, Yunnan 650118, P. R. China; 3 The Second Affiliated Hospital of Kunming Medical University, Kunming, Yunnan 650118, P. R. China

**Keywords:** Brachytherapy, ^125^I seed, Radiation myelopathy, banna mini-pig

## Abstract

**Background:**

Use a banna mini-pig to set up 125I implantation model, and investigate the consequence of radiation-related impairments.

**Methods:**

In present study, ^125^I seeds were implanted into spinal canal of T13 level of spine in banna mini-pigs. After operation, the pigs were raised up to 8 months, behavior changes were recorded within this period. After 8 months, spinal cords were collected for pathological analysis.

**Results:**

In this study, a ^125^I brachytherapy animal model had been successfully established, in the model group, the banna pigs' Tarlov scale decreased from 5 to 2.57 ± 0.36, significant cellular impairments were noted by pathological analysis.

**Conclusions:**

Without any protection and operation improvement, ^125^I implantation can cause serious histological impairments and moving difficulty for banna mini-pigs; this present research provides an alternative tool to study spinal 125I brachytherapy.

## Background

As the most frequent bone metastasis, spinal metastases cause severe pain and damage to vertebral bodies such as spinal osteolytic destruction and compression fractures [1,2].

Brachytherapy, a form of radiotherapy, a radiation source is permanently placed inside or next to treatment locus. Although ^125^I brachytherapy is an effective way to kill tumor cells locally and protect healthy tissues, some complications occurs, such as radiation damages to the tissue around the seeds, which may cause complications, especially radiation myelopathy [3,4]. How to reduce this side effect attracted abundant effort from related medical scientists, but clinically complicate conditions restricted the progress of this kind of research.

Animal model has long been a good tool to help medical researcher to mimic human body situation and find the best way to solve the clinical problems. Aim to solve the radiation myelopathy problem, many rodent animal models had been used, however, the rodent is small in size compared to human and their immunology system is also different from human being, which restricts the usage of this kind of animal model. In this present study, we chose a banna-mini pig as our model animal; because it had similar body size as human and its structure of spinal cord also share the similar anatomy structure with human. Aim to mimic the actual clinical conditions, Digital subtraction angiography (DSA)-guided operation was performed and ^125^I seeds were implanted into spinal canal in T13 level of banna mini-pigs. After raised the banna pigs up to 8 months, the pathological impairments caused by ^125^I were investigated and we also manage to find whether their impairments share the similar character with clinical patients which receive the ^125^I brachytherapy.

## Materials and methods

### Radiation source and reagents

Brachytherapy seeds iodine-125 (BT-125-1) were purchased from Shanghai Xinke Medicine Ltd. Apparent radioactivity was 1.00 mCi/seed and half time is 59.4 days. Before purchase, the ^125^I seeds were randomly picked up for activity testing to confirm the seed container integrity and apparent activity of seeds. In fact, one day before operation, we selected 30 seeds randomly to used hospital setting to test the apparent activity, and found the actual dose per seed was between 0.64 mci-0.67 mci, the associated uncertainty is 0.03 mci, met the requirements.

X-ray computed tomography was from Siemens Company, Germany; DSA (Digital Subtraction Angiography) were from Philips company, Netherlands; Treatment planning system (TPS) were from Hejie medical instrument company, China. CRC-15R calibrator was from CAPINTEC Inc, USA.

All the small instruments including sterile common needle, hammer, seed injection needle were purchase from Guan Long Ltd, Shandong, China.

#### Animal

**Figure 1:**
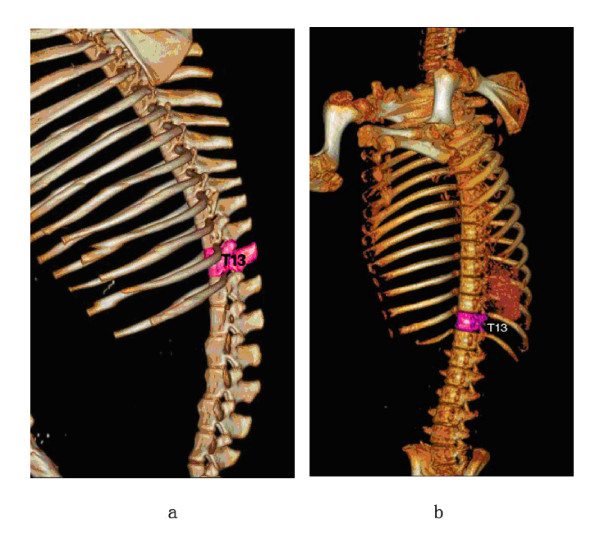
**3D schematic pictures of T13 level of spine in banna mini-pig**.

8 healthy adult female Banna mini-pigs were selected for experiments. The animals were provided and raised by the Animal Center at Kunming Medical College. Their masses ranged from 20 to 25 kg (average 22.7 kg). The mini-pigs adapted laboratory environment for one week before modeling. This housing facility is a barrier housing facility, and it has been in keeping with national standard "Laboratory Animal--Requirements of Environment and Housing Facilities". The care of laboratory animal and the animal experimental operation have conforming to "Chinese Administration Rule of Laboratory Animal", after carful CT scan and consideration, T13 level in spine of banna pig was selected as our ^125^I implanting target because it is easier to control and facilitate the whole operation. The target of T13 level was shown in Figure 1 .

6 pigs were selected to receive the ^125^I implantation, defined as group A, two pigs were kept as age-matched normal control, termed group B. In group A, total 8 brachytherapy seeds were implanted into epidural space outside of vertebral body at T13 level. The pigs were raised up to eight months (equal to 4 half-lives of ^125^I).

### Determination of seed number for implant

Based on the classical dosimetry calculating method cited by Memorial Sloan-Kettering Cancer Center, the seed number was determined by the length of three dimension axis which is the distance from the seed to the target. The formula is following, seed number = [(length + width + thickness/3) ×5] ÷ (seed original activity), and according to the empirical experience, the actual dose density should be increase 20~30% based on the theoretical value.

With help of accurate CT scan, 1 cm is determined for the target length, width and thickness, the apparent activity for one seed is 0.65 mCi, all the data were put into formula, predicted seed number is [(1+1+1)/3 × 5]÷0.65 = 8; the total is approximately 5 mCi.

### Radiation Dose Calculations

This study adopted Monte Carlo-aided dosimetry to measure radiation dose received by mini-pig spine cord in the whole of brachytherapy process, referred from refer "Dosimetry of Interstitial Brachytherapy Sources -- REPORT OF AAPM RADIATION THERAPY COMMITTEE TASK GROUP 43"[5,6]. Briefly, at beginning, we calculate the initial dose (termed as D_(0)_) instantly after the ^125^I particles were implanted into spinal cord T13 level, the formula was D_(0) _= A_0_×1.27 ×Λ×g_(r)_×F_(r θ)_/r^2^, A_0 _is particle initial radiation dose, which was tested by CRC-15R calculator on the day before implanting. Λ is constant parameter for ^125^I, in our research, the value is 1.06, r is the distance between spinal surface to ^125^I particles, which was obtained from the MRI detecting data, g_(r) _is radial dose functions, and F_(r θ) _is anisotropy constant, the detailed data we calculated based on published method [5-7]. After D_0 _has been confirmed, the spine received radiation dose is calculated by formula D_(T)_= D_(0)_*T_1/2_*1.443*[1- e^-T*0.693/T1/2^]. D_(T) _means the total received dose within time interval T, e is natural constant.

#### Operation Procedures

**Figure 2:**
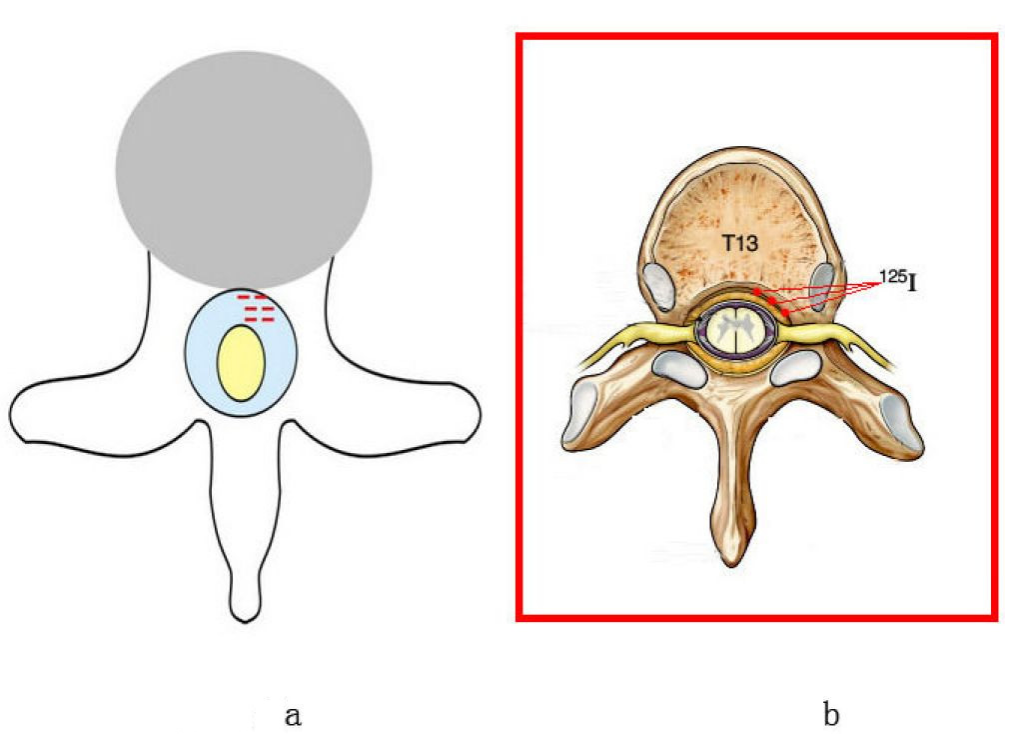
**^125^I seeds implanting Position in spinal canal**.

TPS system was implemented to manage the whole procedure. Before operation, the T13 CT scan photo and physical parameters of ^125^I seeds were put into the TPS system to let the software to create the detailed treatment plan automatically. Then we follow the TPS procedure to do the operation, the mini-pigs were anaesthetized with sodium pentobarbital through ear veins, and then were placed in prone positions followed by skin preparation and sterilization. Digital subtraction angiography was used to precisely localize surface projection of T13 vertebra body and vertebral pedicle. Following the template, a syringe needle, mounted with 20 to 30 degree angle to coronal plate, went into the pedicle of vertebral arch where it connects vertebra, and was placed into the T13 anterior spinal canal. Meglumine diatrizoate was used to confirm the location of the needle. Then ^125^I seeds were implanted into right front of the spinal canal. After the whole operation, CT scan was performed to test the seeds location instantly. Figure 2 showed the detailed implant position in spinal canal.

### Physical condition assessment of model animals

Tarlov scale is a 5-point scale to assess upper and lower limb locomotion. This scale was widely accepted by American Spinal Injury Association (ASIA) as International Standards for assessing the spinal impairment [8]. In this present study, tarlov scale was used to measure the banna-pigs spinal impairment condition after operation.

Hematological analysis were also performed, the peripheral blood sample were collected at five time points, they were before seeds implantation, 1 week, 1 month, 2 month and 8 month post-operation. The white cell number, hemoglobin, blood platelet and creatinine were examined as indexes.

### Pathological analysis

After 8 months, all the pigs were anesthetized and executed, the T13 level was taken out to figure out the distribution about ^125^I seeds. The spinal cord was put into 10% formaldehyde solution for fixation. After 48 hours in 10% formaldehyde solution, spinal cord was cut into 4 mm thick slice for hematoxylin and eosin (HE) staining.

### Double staining of spinal cord for electron microscopy

A series of sections from the vertebral body and spinal cord in 6 banna mini-pigs were processed for electron-microscopic double staining to reveal cellular and sub-cellular alteration in morphology. For this double-labeling procedure, sections were first fixed with 3.5% glutaraldehyde solution and 1% osmic acid solution, and then dehydrated with gradient ethanol and acetone; Last step double stain with uranyl acetate and lead citrate, then observed under JEM-1-11 transmission electron microscopy.

### Data analysis

Standard statistical software(SPSS version15.0; SPSS, Inc, Chicago, IL) was used for data analysis. Student's t-test was used for variable data analysis. P < 0.05 was considered statistically significant. Data were presented at mean ± sd.

## Results

### Confirmation of ^125^I seeds implant at the spinal T13 level by DSA and CT scanning

**Figure 3:**
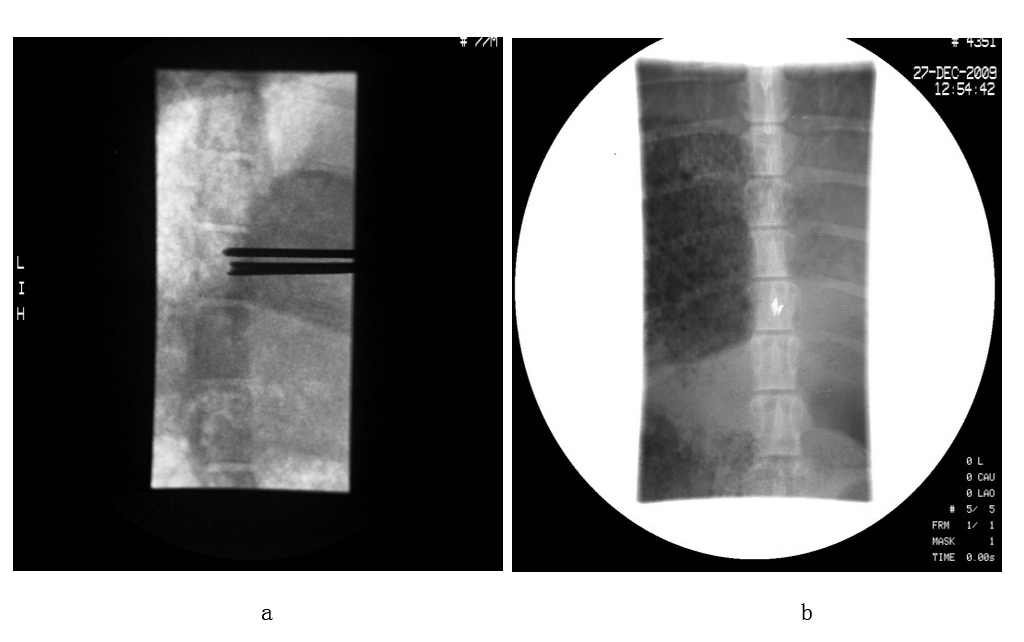
**DSA scan of in-process-operation and post-operation**. A) process of operation, seeds were injecting into T13 level of spine in banna pigs; B) seeds were implanted into target region post-operation.

DSA scan recorded the whole operation procedure, the operation was successfully finished. See Figure 3.

**Figure 4:**
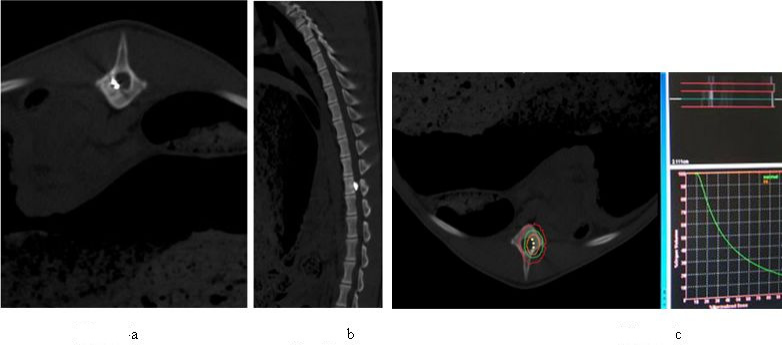
**A) and B) are CT scanning to confirm the location of seeds in T13 level of swine spine; C) is typical isodose and seeds distribution displayed in the cross section CT slices for some individual pig obtained by TPS system**.

After complete the operation 15 minutes, CT scan was performed to confirm the location and distribution of 125I seeds. See Figure 4.

### ^125^I Brachytherapy on Day 0 Post-implant Dosimetry

After operation was completed, CT scan was performed, with the help of TPS, the post-implant dosimetry use the dose-volume histograms (DVH) to assess implant quality, after calculation, minimum peripheral dose (mPD) is 80.1 Gy, D90 is 90.2 Gy, the mean dose for target region is 120.2Gy, D_90 _> mPD, suggested the seeds were successfully implanted into correct position and radiation dose met the requirement of brachytherapy based on the experience from clinical treatment; there is not an acceptable uniform standard for the spinal brachytherapy, this study was also a trial and explosive research, aimed to find the optional seed number for implantation.

### Radiation dose measurement

With CT scan, radiation dose distribution through the axial, sagittal and coronal planes of vertebral body had been determined in this study. Based on the formula mentioned in the "materials and methods" sector, average radiation dose to T13 level vertebral body for each group were obtained, see table 1.

**Table 1:** the parameters of the three dimension of spinal canal and total radiation dose for T13 level

Animals	A (mCi)	Λ	Fan angle	Pitch angle	r	g(r)	f (rq)	Dose rate	D_(4T1/2)_ (cGy)	($\bar{x}$ ± s) (cGy)
1	1.27	1.06	64.2	162.4	0.79	1.039	1	0.91	1752.86	
2	1.27	1.06	64.2	162.4	0.8	1.039	1	0.89	1986.84	
3	1.27	1.06	65.6	160.1	0.8	1.039	1	0.89	1680.38	
4	1.27	1.06	64.5	162.4	0.74	1.039	1	1.04	1972.58	1853 ± 142.89
5	1.27	1.06	65	161.8	0.77	1.039	1	0.96	1986.52	
6	1.27	1.06	63.1	164.1	0.78	1.039	1	0.93	1742.41	

$\bar{x}$ means average dose which vertebral body received in the 8 months.

### Physical condition assessment of Banna mini-pig

No paraplegia case was noted for 8 months after ^125^I implantation, but moving became difficult for banna pigs, two of them couldn't stand up after 2 months. Tarlov scale was 5 for all the normal animals while 2.57 ± 0.36 for experimental animals at 2 months and 8 months later. Hematological analysis didn't find significant change between pre-operation and post-operation, see Table 2.

**Table 2:** hematological index comparison between pre-implantation and post-implantation

Time	N	White cell (X10^9^/L)		hemoglobin(g/L)		platelet (X10^9^/L)		^creatinine ^(μmol/L)	
		
		$\bar{x}$ ± SD	P* value	$\bar{x}$ ± SD	*P value *	$\bar{x}$ ± SD	*P value*	$\bar{x}$ ± SD	*P* *value*
Pre-operation	6	14.23 ± 2.12	_	143.12 ± 4.89	_	452.22 ± 13.06	_	97.71 ± 8.26	_
1 week post implant	6	13.63 ± 1.23	0.475	142.83 ± 4.58	0.412	450.30 ± 16.32	0.327	98.65 ± 7.64	0.263
One month post implant	6	13.85 ± 2.35	0.436	141.57 ± 6.12	0.567	448.12 ± 17.02	0.461	99.01 ± 7.24	0.346
Two months post implant	6	13.84 ± 2.30	0.542	141.57 ± 4.69	0.523	451.98 ± 15.01	0.652	98.97 ± 7.97	0.452
8 months post implant	6	13.697 ± 1.83	0.456	141.79 ± 5.78	0.435	451.57 ± 16.28	0.493	97.05 ± 8.39	0.462

All *P *value was compared with pre-operation data.

### Pathological assessment

**Figure 5:**
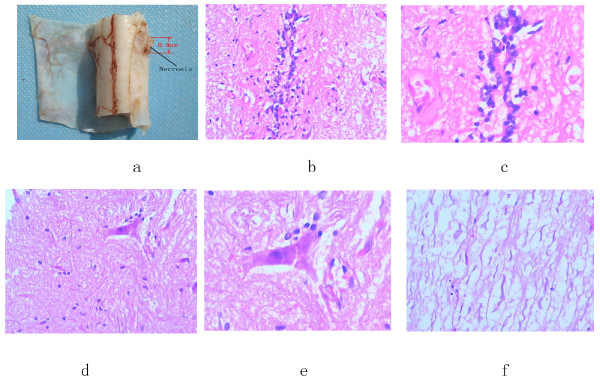
**the pathological analysis of spinal canal**. A) Necrosis region in spinal canal; B) and C) Inflammatory cell invading vessels, HE × 200 and HE × 400 respectively. D) and E) Phagocytosis of neurons, HE stain with × 200 and × 400. F) Demyelination, HE × 200.

The pathological analysis method was referred from widely acceptable procedures [1,9-11]. The obvious liquefaction necrosis was found in the spinal cord by eye observation; the size was around 0.6-0.9 cm, shown in Figure 5a. Under light microscope, inflammatory cells invading the vessels in spinal tissues were noted; grid-like morphological structure change was also found. Necrosis and denature were noted for neurons, axons disappeared, endothelial cells in vessel swelled, nissl bodies dissolved and disappeared, glycogen deposition was found in neurons and micro-vessels. All the pathological changes showed the brachytherapy caused serious cellular impairments for neurons and nerve fibroblasts. See Figure 5.

### Electron-microscopic observations

**Figure 6:**
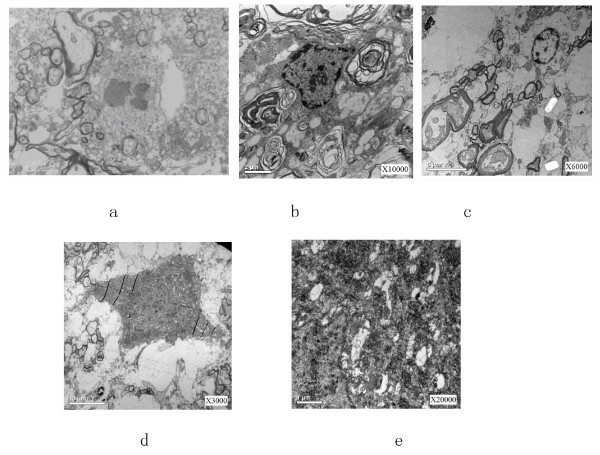
**Electron-microscopic observation A) Axon shrinkage and appearance of apoptotic body; B) Autophagy response; C) and D) shrinkage and swelling of neuron cells; E) Organelles' structure was unclear, mitochondria swelled, nerve fiber dissolved**.

Electron-microscopic (EM) observation was performed for sub-cellular identification. Under EM, dissolving and disappearance happened for nerve fibers, cellular organelles were unclear, obvious impairment was found for most organelles, mitochondria swelled. Apoptosis of neurons was found as well. See Figure 6.

## Discussion

Metastases to the spine are a common problem in a large oncology population. Between 5% and 10% of all cancer patients develop spinal metastases during the course of their disease. Therapeutic intervention can alleviate pain, preserve or improve neurologic function, achieve mechanical stability, optimize local tumor control, and improve quality of life. Treatment options available for metastatic spine tumors include radiation therapy (RT), surgery, and chemotherapy. The appropriate treatment for an individual patient requires a multidisciplinary review including input from a medical oncologist, internist, radiologist, radiation oncologist, neurologist, and surgeon. RT is accepted as the first-line choice for most patients with metastatic spinal tumor.

^125^I brachytherapy was introduced into radiation therapy since 1965[12,13]. Brachytherapy is the placement of radioactive sources in close proximity to any tumor. It takes advantage of the simplest physical properties of radiation. High doses of radiation are present in the vicinity of a radioactive material, and a rapid drop in dose occurs with increasing distance from the source. Brachytherapy has been in use worldwide since shortly after the introduction of radioactive materials. Intracavitary or surface applications are used in some human tumor types [14] (eg, cervix, skin, and bronchus), while interstitial insertions are useful in other situations (eg, head and neck, gynecologic, prostate, and sarcoma[15].

Although ^125^I brachytherapy has been reported to be used in clinical treatment, its mechanism and complication have not been investigated deeply. In this present study, we successfully established a banna mini-pig model to discuss the side effect and pathological influence about ^125^I seeds brachytherapy to neurons and other related cells. Banna mini-pig has similar spinal structure with human, so this exploratory research will be a valuable trial to treat spinal metastasis cancer in animal model.

In this study, all the geometric data related with radiation dosimetry were measured and calculated carefully, guaranteed the radiation accuracy for this whole research, it was most important index for this research. In this study, the average radiation dose which T13 level received met the requirements which simulated the actual clinical situation.

In the clinical treatment, ^125^I can cause radiation myelopathy, in this present study, this point has been confirmed. Moving trouble has been observed for all the operated animals, and by pathological analysis, serious cellular impairments had been found. Electron-microscopic observation gave us further and deep understanding about the extent of impairments. This model provided an effective model to investigate the impair mechanism and it would be a helpful model for researchers to improve the present technique or find a better way to solve the complication.

## Conclusions

Without any protection and operation improvement, 125I implantation can cause serious histological impairments and moving difficulty for banna mini-pigs; this present research provides an alternative tool to study spinal 125I brachytherapy.

## Declaration of interests

The authors declare that they have no competing interests.

## Authors' contributions

ZY was responsible for whole project design and manuscript writing; YX, DY, HS are in charge of establishment of animal model; RZ, XW, and JZ are in charge of data analysis; HJ, LX and JZ are in charge of data analysis and correction of manuscript. All the authors had read and approved the final manuscript.
